# Anisotropic patterns of nanospikes induces anti-biofouling and mechano-bactericidal effects of titanium nanosurfaces with electrical cue

**DOI:** 10.1016/j.mtbio.2024.101352

**Published:** 2024-11-22

**Authors:** Eiji Kato, Masahiro Yamada, Eitoyo Kokubu, Hiroshi Egusa, Kazuyuki Ishihara

**Affiliations:** a Department of Microbiology, Tokyo Dental College, Tokyo, 101-0061, Japan; b Implant & Tissue Engineering Dental Network-Tokyo, 153-0051, Tokyo, Japan; c Division of Molecular and Regenerative Prosthodontics, Tohoku University Graduate School of Dentistry, Sendai, Miyagi, 980-8575, Japan; d Center for Advanced Stem Cell and Regenerative Research, Tohoku University Graduate School of Dentistry, Sendai, Miyagi, 980-8575, Japan

**Keywords:** Bactericidal effects, Contact potential difference, Hydrophilicity, Nanotechnology, Titanium implants

## Abstract

Anti-microbial nanopatterns have attracted considerable attention; however, its principle is not yet fully understood, particularly for inorganic nanopatterns. Titanium nanosurfaces with dense and anisotropically patterned nanospikes regulate biological functions with multiple physical stimulations, which may be because of the nanopattern-induced alternation of surface physical properties. This study aimed to determine the antimicrobial capability of titanium nanosurfaces and their mechanisms. Two types of alkali-etched titanium nanosurfaces with isotropically or anisotropically patterned nanospikes had markedly denser surface protrusions, greater superhydrophilicity, and greater negative charge than machined or micro-roughened titanium surfaces. The crystallographic properties of anisotropic titanium nanosurfaces were similar to those of isotropic nanosurfaces, but markedly higher in electric reactivity at nanoscale. The maximum value of the contact potential difference on titanium surfaces was significantly correlated with the product of the density and anisotropy in the distribution pattern of surface protrusions. Isotropic titanium nanosurfaces did not inhibit the attachment of gram-positive cocci, such as *Staphylococcus aureus*, whereas anisotropic titanium nanosurfaces substantially inhibited gram-positive cocci attachment. Most gram-negative bacilli, *Escherichia coli*, died via swelling of the cell body on anisotropic titanium nanosurfaces within 6 h of incubation, in contrast to other titanium surfaces where most of the cells did not lose viability or undergo morphological changes. The extent of cell swelling was positively correlated with the electric reactivity of the titanium surfaces. Titanium nanosurfaces with anisotropically patterned dense nanospikes exerted anti-biofouling or mechano-bactericidal effects on gram-positive or negative bacteria with electrical cue induced by the anisotropy of the nanospike patterns.

## Highlights

• The physical stimulation–based mechano-bactericidal effects of the titanium oxide nanospikes have attracted attention as a novel anti-microbial principle without any antimicrobial agents.
• Anisotropic nanospike pattern enhances the inherent electric reactivity of titanium surface to induce mechano-bactericidal effects.
• This principle of topographic adjustment may maximally enhance the inherent biological properties of various biomaterials to pave the way for the development of novel implantable biomaterials to simultaneously tune the host/microorganism ecosystem to maintain tissue homeostasis.

## Graphical abstract

**Figure:**
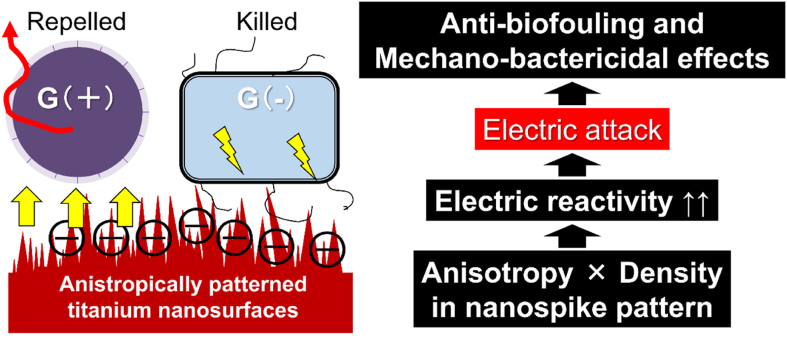

## 1 Introduction

Prevention of microbial infection is crucial for achieving desired outcomes of biomaterial implantation. Tissue-forming cells must win the race against bacteria for colonization and growth on biomaterial surfaces to achieve tissue integration [1,2]. Percutaneous load-bearing devices such as dental or maxillofacial titanium implants require anti-infective control at the tissue–biomaterial interface for a long time after tissue integration [3]. However, the incorporation of anti-infective elements or antibiotics into the biomaterial surface is associated with concerns in the adverse biological effects such as cytotoxicity [4] and the emergence of resistant bacteria [5]. The surface physical properties of biomaterials directly affect bacterial attachment and colonization. Surface hydrophilicity inhibits or promotes bacterial attachment based on the hydrophobic nature of the bacteria [6]. In addition, the surface electric status affects bacterial attachment behavior [7]. Generally, biological cells, including bacteria, are negatively charged; therefore, negatively charged surfaces repel bacteria [6]. Significant increases in surface potential can have bactericidal effects [8,9]. Titanium is covered by a superficial native titanium oxide layer. In the neutral region, the surface potential of titanium oxide is considered to be plus or minus zero [10] or slightly negative [11], indicating that titanium does not inherently exhibit antimicrobial effects. Photoactivation treatment or inorganic elemental coating may alter the titanium surface charge [[12], [13], [14]]. A technology to exert anti-microbial effects by enhancing the inherent surface charge of titanium is required.

Recently, the physical stimulation of the surface nanotopography of biomaterials has attracted attention as a novel anti-microbial principle without any drug or chemical reagents [15]. Nanotopography with nanosized unevenness, which is markedly smaller than bacterial cells, can directly apply various physical stimulations on bacterial cells [15,16]. Nanoprotrusion arrays of organic materials, such as polyethylene terephthalate [17] or polymethyl methacrylate [18], kill *Escherichia coli* by pulling the cell membrane between the protrusions to which the cells attach, and thereby inducing intracellular stress. Among metal-based biomaterials, titanium oxide nanosurfaces are known to exert antimicrobial effects [19,20]. Titanium oxide nanospikes created by alkaline etching treatment have bactericidal potential [21] by physically stabbing bacterial cell membranes [22], similar to the bactericidal nanostructures on the wings of insects such as cicada or dragonfly [15,23,24]. Motile or gram-negative bacteria may be more sensitive to the physical stimulation–based bactericidal effects of titanium oxide nanosurfaces [22,25]. The generation of intracellular oxidative stress may induce the physical stimulation–based bactericidal effects of titanium oxide nanospikes [26]. However, the principles of the physical stimulation–based mechano-bactericidal effects of titanium oxide nanospikes have not been elucidated.

Surface roughness affects the electrical properties of material surfaces [27]. The distribution of electric potential and range of electric field strength are affected by the surface topography of the plasma-facing components [28]. A roughened titanium surface with sharp edges or vertices has increased surface charge density and electric field strength at the tips of the edges or vertices [29]. For instance, the distribution profile between the vertex shape and surface potential matches on the titanium surface coated with calcium phosphate by radio-frequency magnetron sputtering [30]. Nanoscale tuning of titanium surface topography allows for more effective control of the surface electric potential [31]. Moreover, the charge distribution state affects the electrical reactivity in the configuration space at the nano scale or lower. Intramolecular anisotropy of charge distribution drives noncovalent interactions between molecules [32]. The anisotropic electron distribution within the chlorine atom of carbon tetrachloride induces intermolecular electron transfer as the driving force for halogen bond formation [33]. This suggests that in the nano-scale configuration, anisotropy of surface electric potential distribution can enhance electric reactivity.

Based on this information, we hypothesized that increased anisotropy in the nanovertex distribution pattern enhances surface electrical reactivity, thereby strengthening the antibacterial activity of titanium nanosurfaces. The purpose of this study was to investigate the electrical reactivity and antibacterial properties of titanium nanosurfaces with anisotropically patterned and dense nanospikes created by alkaline etching.

## 2 Material and methods

### 2.1 Preparation of titanium samples

Commercially pure grade II titanium discs (10-mm diameter, 1-mm thick) and grade I titanium sheets (14.0-mm square, 0.18-mm thick) were purchased from Nishimura Co., Ltd. (Fukui, Japan). The as-prepared titanium samples with machined surfaces (MA) were washed by ultrasonication in a series of ethanol and distilled water (DW) solutions after acetone cleaning. Titanium samples with acid-etched surfaces were prepared by immersion of the cleaned MA titanium samples in 67 % (w/w) sulfuric acid solution (FUJIMA Wako Pure Chemical Corporation, Osaka, Japan) at 120 °C for 75 s and used as representative micro-roughed (MR) surfaces. Two types of nano-roughened (NR) titanium surfaces, namely, NR_iso and NR_ani, respectively, were prepared based on previously reported alkaline etching protocols [34,35]. The detailed methodology for nanosurface modification has been shown in supplementary material and methods. All samples were sterilized by autoclaving immediately before the experiment.

### 2.2 Scanning electron microscope analyses on surface topography

The surface topography of the titanium surfaces was evaluated by scanning electron microscopy (SEM) using a model JSM-6390LA microscope (JEOL Ltd., Tokyo, Japan) and a model XL30 microscope (Philips, Eindhoven, the Netherlands), and by laser microscopy using a Talysurf PGI 1250A microscope (AMETEK Taylor Hobson, Leicester, UK). Elemental analysis of the titanium surfaces was performed using an EX-94300S4L1Q energy-dispersive X-ray spectrometer (EDX) (JEOL Ltd.) incorporated into the SEM. The horizontal pattern and distribution of spikes on the MR and NR surfaces were evaluated on their vertex extraction images from secondary electron SEM images using a WinRoof image analyzer (MITANI Corporation, Tokyo, Japan) as previously described [36,37]. Vertex density was calculated by measuring the number of vertices per square micrometer. Based on the centroidal Voronoi tessellation, Voronoi diagrams of the vertex-extracted images were prepared by dividing the area such that the vertex of each spike became the centroid of the area. The anisotropy in the vertex distribution pattern was calculated by dividing the mean of the tessellated areas on each surface by the corresponding standard deviation. To evaluate the size of the pits on each titanium surface, vertex-extracted images were analyzed using the center-of-gravity method to create tessellated areas by connecting the three closest points to the center of gravity of the vertexes. The Feret diameters of the tessellated areas were regarded as the size of the pits on each titanium surface. The vertical roughness parameters of the arithmetical mean height (Sa) and maximum valley depth (Sv) were measured at a measurement length of 50 μm on each titanium surface using the laser microscope after removing waviness by approximating a cubic polynomial.

### 2.3 Transmission electron microscopy (TEM) analysis

Ultrathin longitudinal sections of NR_iso and NR_ani titanium discs were prepared using the ion-milling method for metal specimens, as previously described [36,37]. Briefly, NR_iso and NR_ani titanium discs were bonded with a dummy lining material using epoxy resin, shaped using a cutting machine, thinned along the longitudinal sectional direction using mechanical polishing, and ultrathinned on a ﬁxing mesh using a precision ion polishing system (PIPS) 691 ion-milling machine (Gatan, Pleasanton, CA, USA). Bright-field observations and elemental analyses of ultrathin longitudinal titanium sections were performed by transmission electron microscopy using a model HF-2000 microscope system (Hitachi High-Tech Corporation, Tokyo, Japan) at an acceleration voltage of 200 keV.

### 2.4 Grazing incidence X-ray diffraction analysis

The crystalline phase of each titanium surface was analyzed using grazing incidence X-ray diffraction (GIXRD). Irradiation of the sample with X-ray-beam at a very low angle of incidence enabled qualitative analysis of the crystalline phase present in the top few nanometers of the material. Titanium discs were analyzed using a Θ/Θ-diffractometer (SmartLab, Rigaku Co., Ltd., Tokyo, Japan) with the CuKα radiation (λ = 1.5418 Å, U = 45 kV, I = 200 mA) at 0.4° incidence angle. The crystalline phase was determined by cross-checking the measurement patterns with a powder diffraction file (International Centre for Diffraction Data) using a search-and-match algorithm.

### 2.5 Wettability

To evaluate the titanium surface energy, contact angles for 10-μL DW droplets were measured on each titanium surface using a CA-X sessile drop machine (Kyowa Interface Science Co. Ltd., Saitama, Japan), following the θ/2 method. Water contact angles >90°, <90°, and <10° were defined as hydrophobic, hydrophilic, and superhydrophilic, respectively [36].

### 2.6 FTIR spectroscopy

Functional groups on the titanium surface were analyzed using Fourier transform infrared (FTIR) spectroscopy using an IRT7000 linear array imaging microscope (JASCO Corporation, Tokyo, Japan). Micro-reflection spectra were recorded in the range of 4000–2000 cm^−1^ at a spectral resolution of 4 cm^−1^ with 500 accumulations and a 50-μm^2^ aperture. Background correction was performed based on the surface spectrum of MA.

### 2.7 Zeta potential and particle size measurements

To evaluate the electrical properties of the NR_iso and NR_ani titanium surfaces, the isoelectric point and zeta potential of the respective sheets were analyzed using a zeta-potential analyzer ELS-Z2 (Otsuka Electronics Co., Ltd., Osaka, Japan). The pH titration plot of a nano-particle suspension of material comprising a superficial layer of NR_iso or NR_ani titanium sheets was drawn to determine the isoelectric point by measuring the zeta potential at each pH value. Electro-osmotic flow of the monitored colloidal solution on the titanium sheets was analyzed to directly evaluate the zeta potential of the titanium surface with an individual vertex distribution. The detailed methodology has been shown in supplementary material and methods. All samples were sterilized by autoclaving immediately before the experiment.

### 2.8 Kelvin probe force microscopic analysis

The MA, NR_iso, and NR_ani titanium discs were analyzed using a Dimension icon XR scanning probe microscope (Bruker Corporation, Billerica, MA, USA) in the Kelvin probe force microscopy (KPFM) mode to evaluate the surface potential distribution and electrochemical reactivity of the titanium nanosurfaces. The titanium surfaces were scanned over a 1-μm square area at a resolution of 256 pixels square using a silicon probe in peak force tapping mode combined with frequency modulation feedback. In correspondence with the topological image, the surface potential image was obtained, depicting contact potential differences based on the difference in the work function that occurs when the silicon probe is in electrical contact with the titanium surface. Contact potential differences were quantified in each 15-nm square, excluding outliers owing to tip-shape effects.

### 2.9 Bacterial strain and culture condition

*Staphylococcus aureus* 209P, *Streptococcus sanguinis* ATCC 10556 and *E. coli* Top 10 (ThermoFisher Scientific, Waltham, MA, USA) were used in the experiments. *S. aureus* was grown in 3.0 mL brain heart infusion broth (BHI, Becton Dickinson, Sparks, MD, USA) in a 12-mL sterile plastic tube capped and statically incubated at 37 °C overnight (16–18 h). *E. coli* was incubated with shaking at 37 °C overnight. S. sanguinis was statically incubated anaerobically (N2: 80％, H2: 10％ and CO2: 10％) at 37 °C overnight. Contamination by any other bacteria was ruled out with Gram staining and the optical density (660 nm) of the bacterial suspensions was adjusted to 0.02, 0.1, or 0.5 using a multi-mode microplate reader prior to experimental use.

From each culture, 1 mL of suspension in BHI or phosphate-buffered saline (PBS) (Cat# 05913, Shimadzu Diagnostics Corporation, Tokyo, Japan) was placed onto titanium discs in a sterilized 12-well polystyrene culture plate. As a result of preliminary investigation of the culture conditions, titanium discs were incubated in *S. aureus* suspension adjusted to an optical density of 0.1 for up to 8 h in a humidified atmosphere of 95 % air at 37 °C (Fig. S1). The *E. coli* suspension was incubated with titanium discs in the same atmosphere for 1, 3, and 6 h. The *S. sanguinis* suspension was incubated for 2 h anaerobically mentioned above. After incubation, the titanium discs were removed from the bacterial culture using sterilized forceps and gently rinsed in PBS to remove planktonic cells. The discs were transferred to sterilized polystyrene dishes for microbial detection.

### 2.10 Evaluation of bacterial attachment or biofilm formation on titanium surface

After incubation in the bacterial suspension, the titanium discs were washed twice with PBS and prefixed with a 10 % buffered neutral formalin solution (Code 20211; Muto Pure Chemicals Co., Ltd. Tokyo, Japan) at 4 °C for 1 h. After washing with PBS, the discs underwent postfixation with 2 % glutaraldehyde solution (Code: 071-02031; Wako Pure Chemical industries, Ltd, Osaka, Japan) at 4 °C for more than 3 h. After fixation, the discs were washed with distilled water and dehydrated in an ethanol series for 10 min at each step. After substitution with t-butyl alcohol (Code: 028-03386; Wako), t-butyl alcohol was sublimated using a freeze dryer (VFD-30; Vacuum Device Inc., Ibaraki, Japan). After sputter coating with a gold/palladium alloy, bacterial colonization on the titanium surfaces was observed using SEM with a model XL30 microscope. From the secondary electron image at 10,000× magnification, the density and morphological characteristics (area and Feret diameter) of bacteria attached to the surface were measured using ImageJ software version 1.53t (National Institutes of Health, Bethesda, MD, USA).

The metabolic activity of bacteria on titanium surfaces was quantified by a bioluminescence-based adenosine triphosphate (ATP) assay using BacTiter-Glo Reagent (Promega Corporation, Madison, WI, USA). The assay was based on the principle that luminescent signals were generated through mono-oxygenation of luciferin catalyzed by luciferase in the presence of magnesium ion, ATP and molecular oxygen. After 2-, 3-, or 8-h incubation, 500 μl of the reagents were added to the PBS containing the cultured disc and luminescent intensity was measured using a microplate reader (SpectraMax M5^e^, Molecular Devices, Tokyo, Japan) according to the manufacturer's instructions.

Biofilms formed by *S. aureus* after 8 h of incubation on titanium surfaces were stained with 1 mL of 0.1 % crystal violet (Cat#, company name) for 15 min. After washing with distilled water, the biomass-associated crystal violet was extracted with 1 mL 99.5 % ethanol. The extracts were transferred to a new microtiter plate and the optical density was measured at 595 nm using a microplate reader.

### 2.11 Live and dead analysis

To evaluate the viability of bacteria on the titanium surfaces after 2 or 3 h of incubation, the bacteria on the titanium discs were incubated with live and dead staining (Live/Dead BacLight Bacterial Viability Kit, Molecular Probes, Eugene, OR, USA) in the dark at room temperature for 15 min according to manufacturer's instructions. After washing twice with PBS, the titanium discs were observed using a confocal laser scanning microscope (LSM5 DUO; Carl Zeiss MicroImaging Inc., Göttingen, Germany). Colonized bacteria were scanned at excitations of 488 nm and 543 nm to label cells as viable and dead cells, respectively. The area ratio of live or dead cells to the total area of the visual field was calculated as a percentage of live or dead cells.

### 2.12 Evaluation of protein absorption on titanium surface

To evaluate protein absorption from the culture media onto titanium surface, 500 μL of BHI without bacteria was placed on titanium discs in 24-well culture-graded polystyrene culture plates and incubated in a humidified atmosphere of 95 % air and 5 % CO_2_ at 37 °C for 8 h. After incubation, the culture media on the polystyrene culture plates were discarded and the titanium discs were transferred to a new culture plate. Reagents for detection of tripeptides, polypeptides, and proteins with three or more amino acids (PierceTM BCA Protein Assay Kit, Thermo Fisher Scientific) were placed in the culture plate and incubated for 30 min at 37 °C. The resulting purple solution was transferred to a new microtiter plate, and the optical density was measured at 562 nm using a microplate reader.

### 2.13 Statistical analysis

Three independent samples of each titanium surface were subjected to a series of quantitative measurements for surface topography, zeta potential of the flat surface, and protein absorption (*N* = 3). In image analyses, the mean value from three randomly selected areas on the surface was calculated for each sample value. The Feret diameters of the pits were analyzed, including all detected pits in the representative SEM images of the three samples on each substrate (*N* = 209-2,078). For EDX, TEM, FTIR, KPFM, and zeta potential analyses of the particles, multiple samples were analyzed. Data from the representative samples are presented. Contact potential differences in KPFM were analyzed every 15-nm square within a representative 1-μm square area, and the mean and unbiased variance were calculated on each set (*N* = 9,356–9,362). All culture experiments were performed in at least three independent cell batches (*N* = 3-15). The bacterial density of *S. aureus* was analyzed using randomly selected SEM images of multiple cultures on each substrate (*N* = 10-17). Cytomorphometry of *E. coli* shapes on titanium surfaces was performed on single cells in randomly selected SEM images of multiple cultures on each substrate (*N* = 46-193). Immunofluorescent analyses for live and dead staining were performed at least in ten randomly selected confocal laser microscopic images of multiple cultures on each substrate (*N* = 10-17). One-way analysis of variance (ANOVA) was used to assess differences among multiple experimental groups. When appropriate, a post hoc Dunnett's test, Tukey's honest significant difference (HSD) test, Bonferroni multiple comparison test, or Games–Howell test was used. The Spearman rank-order correlation test was used to assess the correlation between the maximum value of the contact potential difference or the average zeta potential of the solid surface, and the product of the anisotropy and density of the vertex on each titanium surface and between the average value of the water contact angle or zeta potential of the solid surface and the average luciferase activity of *S. aureus* in BHI for 2 h. *P* < 0.05 was considered statistically significant. All statistical analyses were performed using the JMP Pro software. 17 (SAS Institute Japan Ltd., Tokyo, Japan).

## 3 Results

### 3.1 Topographic characteristics of titanium nanosurfaces

**Fig. 1:**
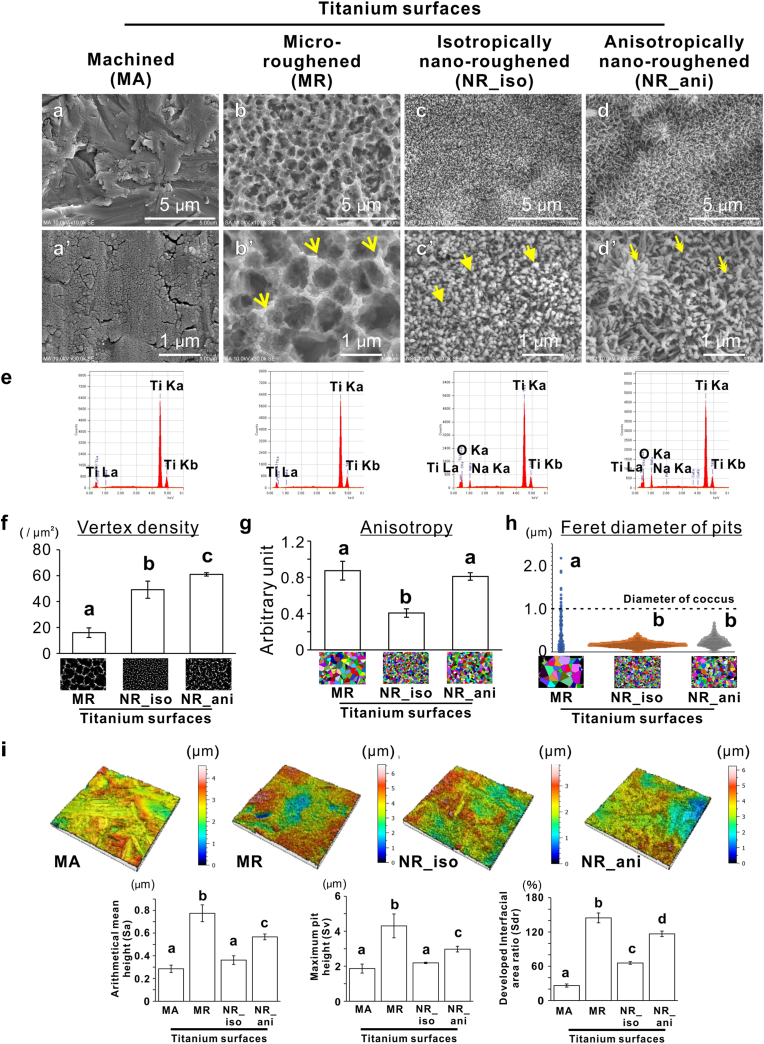
Topographic characteristics of titanium nanosurface, Representative secondary scanning electron microscopy images of titanium discs with (a) machined (MA), (b) micro-roughened (MR), (c) isotropically nanoroughened (NR_iso), and (c) anisotropically nanoroughened (NR_ani) surfaces. Higher magnification images of each corresponding surface are presented in the lower panels (a–d). (e) Energy-dispersive X-ray spectroscopy profile of each titanium surface. Vertex density (f), anisotropy (g) in vertex patterns, and Feret diameter of pits (h) of MR, NR_iso, and NR_ani surfaces together with the corresponding typical analyzed images of vertex extraction and Voronoi diagrams drawn using Voronoi tessellation and the center-of-gravity method, respectively. Vertical roughness parameters (i) of arithmetical mean height (Sa), maximum pit height (Sp), and developed interfacial area ratio (Sdr) with representative three-dimensional height bird's eye view images. Data are presented as means ± standard deviation (SD) (*N* = 3) in (f), (g), and (i), and dot plots (*N* = 209-2078) in (h). Different letters indicate statistically significant differences (*P* < 0.05; Tukey's honestly significant differences test). The dashed line in (h) indicates the general diameter of the cocci cells. Note: sharp ridges and numerous micro-pits are evident on the MR titanium surface (arrows in b’). Multiple nanospikes are observed on both the NR_iso and NR_ani titanium surfaces, but with greater randomness on the NR_ani surface (arrowheads in c’ and double arrowheads in d’).

Secondary electron SEM images showed flat polishing marks (Fig. 1a–a’) and micron-sized ridges and pits (Fig. 1b–b’, arrows) on the MA and MR titanium surfaces, respectively. Both types of titanium nanosurfaces exhibited dense and numerous nanosized spikes. However, the NR_iso surfaces had relatively isotropically distributed nanospikes (Fig. 1c–c’, arrowheads), whereas the NR_ani surfaces had anisotropically distributed nanospikes that tended to be crowded in uneven locations (Fig. 1d–d’, double arrowheads). The EDX analysis detected only titanium on the MA and MR surfaces. In addition, oxygen and sodium were also detected on the nanosurface (Fig. 1e). In contrast, the titanium surfaces treated with 5 or 10 M alkaline etching showed numerous nanospikes composed of titanium, oxygen, and sodium (Fig. 1e). The vertex densities of the NR_iso and 2 surfaces were two and three times higher, respectively, than that of the MR surface (Fig. 1f). Anisotropy in the two-dimensional distribution of vertices was low on the NR_iso surface but high on the MR and NR_ani surfaces (Fig. 1g). Feret diameter of pits between nanospikes on both types of titanium nanosurfaces were smaller than 1.0 μm (Fig. 1h).

The MR surfaces exhibited the highest vertical roughness values, such as arithmetic mean height (Sa) and maximum pit height (Sv), and the surface area increased by up to 140 % of the developed interfacial area ratio (Sdr) (Fig. 1i). The NR_iso surface did not show increased the vertical roughness value compared to that of the MA surfaces but showed increased Sdr by up to 60 %.

### 3.2 Crystallographic and electric properties of titanium nanosurfaces

**Fig. 2:**
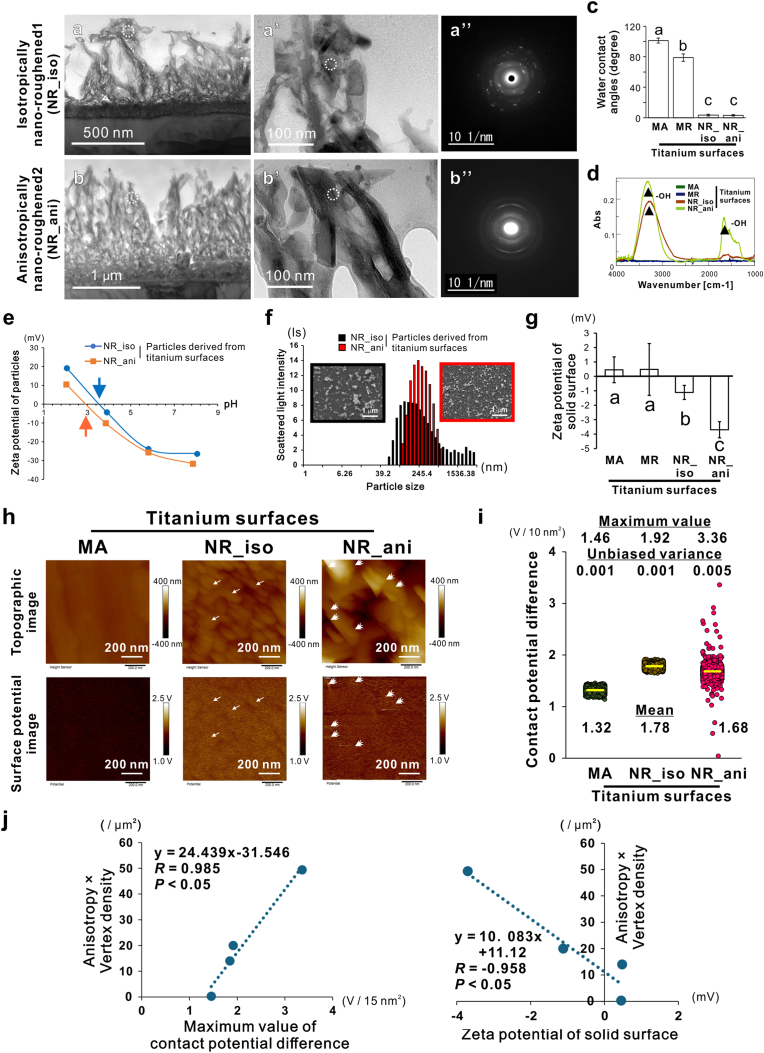
Crystallographic and electric peripeties of titanium nanosurface, Bright-field and selected-area electron diffraction images (a and b) of ultrathin titanium longitudinal sections of isotropically nanoroughened (NR_iso) (a-a”) and anisotropically nanoroughened (NR_ani) (b-b”) titanium surfaces were examined using transmission electron microscopy. Data from each dashed circular spot show the corresponding typical features of the superficial layer. Water contact angles (c) and Fourier transform infrared (FTIR) spectra (d) on each type of titanium surface. Triangles on FTIR spectra (d) indicate peaks corresponding to hydroxyl groups (-OH). (e) Titration plots and (f) particle size distribution of representative scanning electron microscopic images of exfoliated nanoparticles from NR_iso and NR_ani titanium nanosurfaces. Zeta potential of the solid surface (g) of each titanium sample. Representative topographic and surface potential images (h) and contact potential differences (i) evaluated using Kelvin probe force microscopy on each titanium surface. Scatter plots (j) showing the correlation between the product of the anisotropy and density of the vertex and the maximum value of the contact potential difference or the average zeta potential of the solid surface on each titanium surface. Data are presented as means ± standard deviation (SD) (*N* = 3) in (c) and (g), and dot plots (*N* = 9,356–9,362) with mean (yellow line), unbiased variance, and maximum value in (i). Different letters indicate statistically significant differences (*P* < 0.05; Tukey's honestly significant differences test). Note: Vertices correspond to points of high contact potential difference, and the intensity and distribution of surface potentials are uneven on the NR_ani titanium surface (double arrowheads in h), in contrast with the even distribution on the NR_iso surface (arrowheads in h).

Both types of titanium nanosurfaces consisted of a spongy and shaggy superficial layer with some channels transitioning from a dense transitional layer to a titanium base (Fig. 2a and b). According to the SEM analysis and vertical roughness values, the density and thickness of the superficial layer were greater on the NR_ani surface than on the NR_iso surface. Thickness of the superficial layer was 500–700 nm and approximately 1.0 μm on the NR_iso and 2 surfaces, respectively. However, the crystallographic, hydrophilic, and chemical properties of the NR_iso and NR_ani surfaces were similar, showing an amorphous-like crystalline structure oriented toward the outermost side (Fig. 2a’ and **b’**) and a lattice space matching brookite-type titanium oxide in the electron diffraction images (Fig. 2a’’ and **b’’**). GIXRD revealed that the XRD patterns were similar for NR_iso and NR_ani surfaces, which contained peaks indicating titanium dioxide and sodium titanate, in contrast to only titanium detected on the MA surface (Fig. S2).

The NR_iso and 2 titanium surfaces were superhydrophilic at several degrees in contrast with the sessile drop-based water contact angle of 90 and 80° on the MA and MR titanium surfaces (Fig. 2c). In addition, hydroxyl groups were detected on the NR_iso and 2 titanium surfaces, but not on the MA and MR titanium surfaces, as determined using Fourier transform infrared spectroscopy (Fig. 2d).

Isoelectric points of particles obtained by exfoliating the titanium nanosurfaces were 3.5 and 3.0 on the NR_iso- and NR_ani-derived particles, respectively (Fig. 2e). Changing profile of zeta potentials by an increase of pH was similar between the NR_iso- and NR_ani-derived particles, and both particles showed a potential of approximately −25 mV at pH 5.8 (Fig. 2e). The NR_iso-derived particles were 790.8 nm (76.8–5900 nm or more) in the average particle size, which was consistently larger than the NR_ani-derived particles with 215.6 nm (98.1–614 nm) (Fig. 2f). Likewise, the zeta potential of the solid surface (pH 6.0) was negative for both types of titanium nanosurfaces, in contrast with approximately 0 mV on the MA and MR titanium surfaces (Fig. 2g). However, the NR_ani titanium surface showed a zeta potential 3.7 times more negative than that of the NR_iso surface (Fig. 2g).

KPFM analysis showed that the increase in the contact potential difference with the probe coincided with the vertices of the spikes and ridges for all titanium surfaces (Fig. 2h, white arrowheads). The mean value of the NR_ani titanium surface was not greater than that of the NR_iso titanium surface. However, the maximum value reached over 3.36 V on the NR_ani titanium surface in contrast to less than 2.0 V on the other titanium surfaces (Fig. 2i). In addition, the variation in the contact potential difference was markedly increased only on the NR_ani titanium surfaces, where the unbiased variance was five times higher than that on the MA and NR_iso titanium surfaces (Fig. 2i). The product of the distribution anisotropy and density of the surface vertices positively correlated with the maximum contact potential difference and negative zeta potential values (Fig. 2j).

### 3.3 Effects of titanium nanosurfaces on gram-positive cocci colonization

**Fig. 3:**
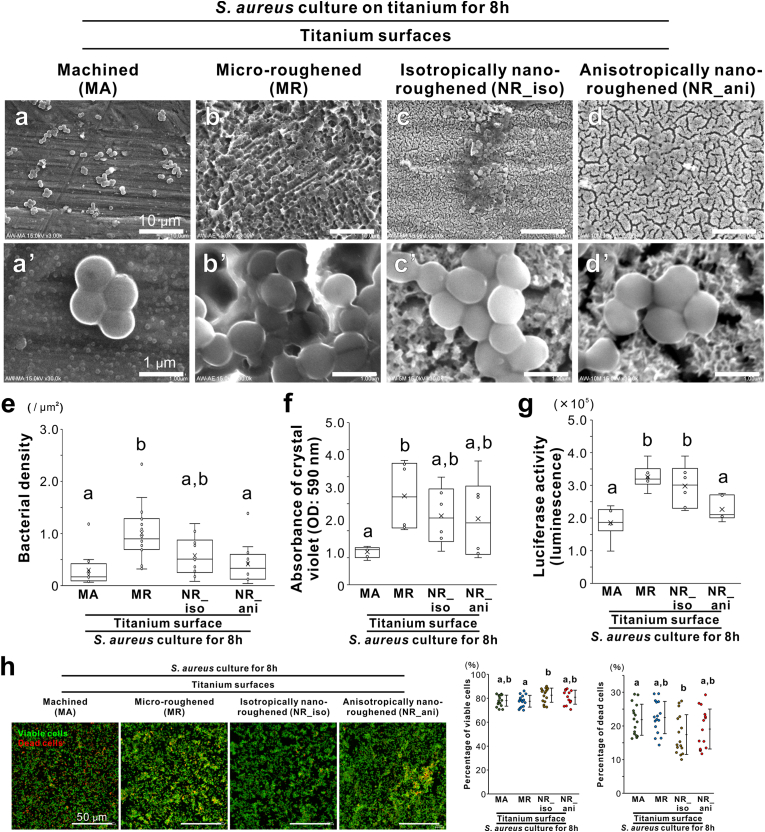
Effects of titanium nanosurface on *Staphylococcus aureus* colonization and viability, Scanning electron microscopy (SEM) images of *Staphylococcus aureus* 209P strain cultured in brain heart infusion broth for 8 h on machined (MA) (a), micro-roughened (MR) (b), nano-roughened 1 (NR_iso) (c), and nano-roughened 2 (NR_ani) (d) titanium surfaces. Higher magnification images of each corresponding culture are presented in the lower panels (a–d). Bacterial density was analyzed using SEM images (e), absorbance of crystal violet staining (f), luciferase activity measuring the amount of bacterial adenosine triphosphate (g), confocal laser microscopy images, and quantification of the percentages of viable and dead cells after live and dead fluorescent staining (h) in the bacterial culture for 8 h on each titanium surface. Data are presented as box-and-whisker plots with dots and mean ( × ) in (e) (*N* = 10–16), (f), and (g) (*N* = 6), and dot plots (*N* = 14–16) with mean (yellow line), unbiased variance, and maximum value in (i). Different letters indicate statistically significant differences (*P* < 0.05; Tukey's honestly significant differences test or Bonferroni multiple comparison test).

SEM observations of *S. aureus* culture on the titanium surfaces for 8 h in BHI broth indicated that the cocci were trapped and grew in recesses on the MR surfaces (Fig. 3b). In contrast, the cells simply attached to the MA surfaces (Fig. 3a), and both NR surfaces with indentations smaller than the cell body showed a similar colonization appearance (Fig. 3c and d). The MR surface had the highest bacterial density, as counted using SEM, and most intense crystal violet staining for the 8-h bacterial culture, whereas both NR surfaces had values comparable with the MA surfaces (Fig. 3e and f). The luciferase activity indicated that the ATP concentration in the colonized bacteria was higher on the MR and NR_iso surfaces than on the MA and NR_ani surfaces (Fig. 3g). Live and dead staining for the 8-h cultures showed that the development of colonized bacteria was apparently greater on the MR surfaces than on the other surfaces (Fig. 3h). However, the percentages of viable and dead cells in the attached bacteria were comparable among the surfaces, although the colonized bacteria on the NR_iso titanium surfaces tended to be more viable and had fewer dead cells (Fig. 3h).

### 3.4 Effects of titanium nanosurfaces on initial attachment of gram-positive cocci

**Fig. 4:**
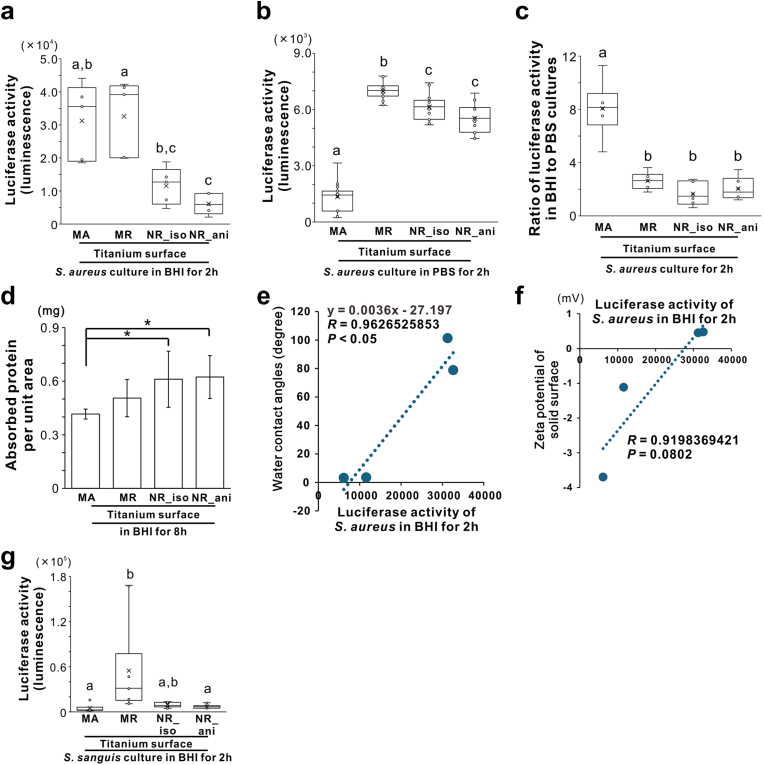
Effects of titanium nanosurface and associated surface physical properties on initial attachment of gram-positive cocci, Luciferase activity measuring the amount of bacterial adenosine triphosphate in *Staphylococcus aureus* 209P strain culture in brain heart infusion (BHI) broth (a) or phosphate-buffered saline (PBS) (b) for 2 h, the ratio of luciferase activity in BHI and PBS (c), and absorbed protein per unit area after 8-h incubation (d), on machined (MA), micro-roughened (MR), nano-roughened 1 (NR_iso) and nano-roughened 2 (NR_ani) titanium surfaces. Scatter plots show the correlation for each titanium surface between the average luciferase activity of *S. aureus* in BHI for 2 h and the average water contact angle (e) or zeta potential of the solid surface (f) of each titanium surface. Luciferase activity of *Streptococcus sanguinis* ATCC 10556 strain in the BHI broth for 2 h (g). Data are presented as box-and-whisker plots with dots and mean ( × ) in (a) (*N* = 5), (b) (*N* = 15), (c) (*N* = 6), and (g) (*N* = 10), and means ± standard deviation (SD) (*N* = 4) (d). Different letters or asterisks indicate statistically significant differences (*P* < 0.05; Tukey's honestly significant differences test, Bonferroni multiple comparison test, Games–Howell test or Dunnett's test).

Luciferase activity in the 2-h *S. aureus* culture in BHI was the lowest on the NR_ani titanium surfaces (Fig. 4a). In addition, the activity on the NR_iso surface was lower than that on the MR surface and comparable to that on the MA surface (Fig. 4a). In contrast, the 2-h bacterial culture in PBS showed the lowest luciferase activity on the MA surfaces (Fig. 4b). Although much higher than that on the MA surfaces, the NR_iso and NR_ani surfaces had consistently lower values than the MR surfaces (Fig. 4b). The ratio of luciferase activity in the BHI to PBS culture was four times higher on the MA surface than on the other surfaces (Fig. 4c). The amount of BHI protein adsorbed was higher on the NR_iso and NR_ani titanium surfaces than on the MA surfaces (Fig. 4d). The water contact angle positively correlated with the luciferase activity of the 2-h *S. aureus* culture in BHI on the titanium surfaces (Fig. 4e). In addition, the negative zeta potential was positively correlated with the initial *S. aureus* attachment on the titanium surfaces (Fig. 4f). Both types of nanoroughened titanium surfaces reduced the initial attachment of another genus of gram-positive cocci, *S*. *sanguinis*, to a level equivalent to that on MA surfaces (Fig. 4f).

### 3.5 Effects of titanium nanosurfaces on morphological changes of gram-negative bacilli

**Fig. 5:**
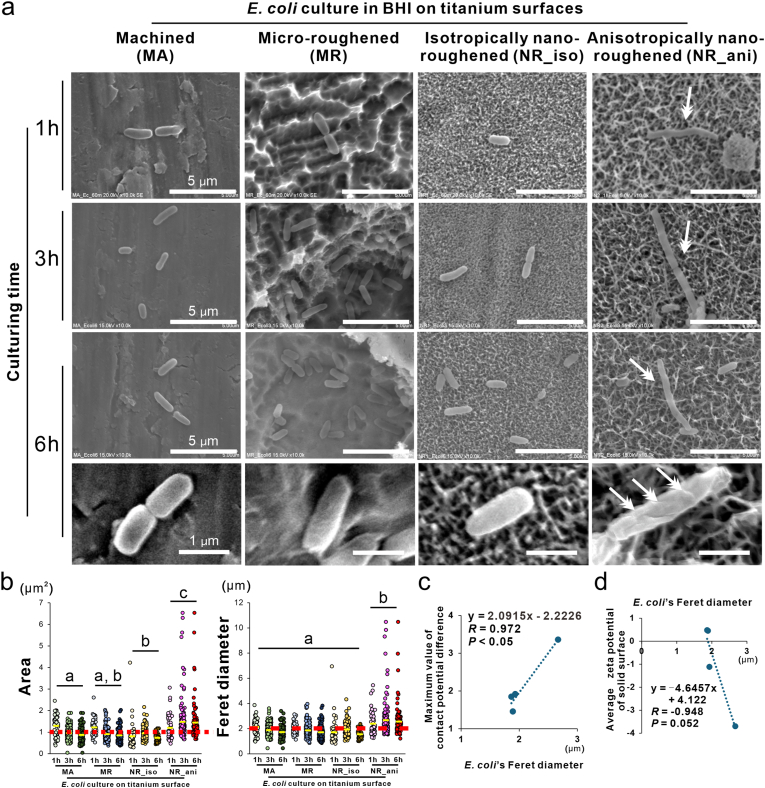
Effects of titanium nanosurface and associated surface physical properties on morphological changes of *Escherichia coli,* Scanning electron microscopy (SEM) images (a) of *Escherichia coli* Top10 strain cultured in brain heart infusion broth for 1, 3, and 6 h on machined (MA), micro-roughened (MR), nano-roughened 1 (NR_iso) and nano-roughened 2 (NR_ani) titanium surfaces. Higher magnification images of each corresponding culture are presented in the lower panels (a–d). The bacterial cell area and Feret diameter were analyzed from SEM images after 1-, 3-, and 6-h incubation on each titanium surface (b). Scatter plots showing the correlation between the average Feret diameter of *E. coli* in BHI for 3 h and the maximum value of the contact potential difference (c) or average zeta potential of the solid surface on each titanium surface. Data are presented as dotted plots (*N* = 46-193) with the mean (yellow line) and general lengths of *E*. *coli* (dashed red line). Different letters indicate statistically significant differences (*P* < 0.05; Tukey's honestly significant differences test). Note: Elongated *E. coli* with crumpled cell surface was observed only on the NR_ani titanium surface throughout the 6-h incubation period (double arrows in a).

Under SEM observation, *E. coli* showed an elongated and crumpled cell body on the NR_ani titanium surfaces throughout the culture period from 1 to 6 h, whereas other titanium surfaces had normal-shaped cells (Fig. 5a). The area and Feret diameter of the cell body were consistently higher on the NR_ani surfaces than on the other surfaces over the 6-h culture period (Fig. 5b). The average values of the area and Feret diameter on the NR_ani surfaces were higher than the general size of *E coli* [38], whereas the average values on the other surfaces were not beyond the general size (Fig. 5b). The Feret diameter of *E. coli* on titanium surfaces was positively correlated with the electrical properties of the titanium surfaces, particularly with the maximum contact potential difference (Fig. 5c). The more negative the zeta potential of the titanium surface, the longer the *E. coli* tended to become (Fig. 5d).

### 3.6 Effects of titanium nanosurfaces on viability of gram-negative bacilli

**Fig. 6:**
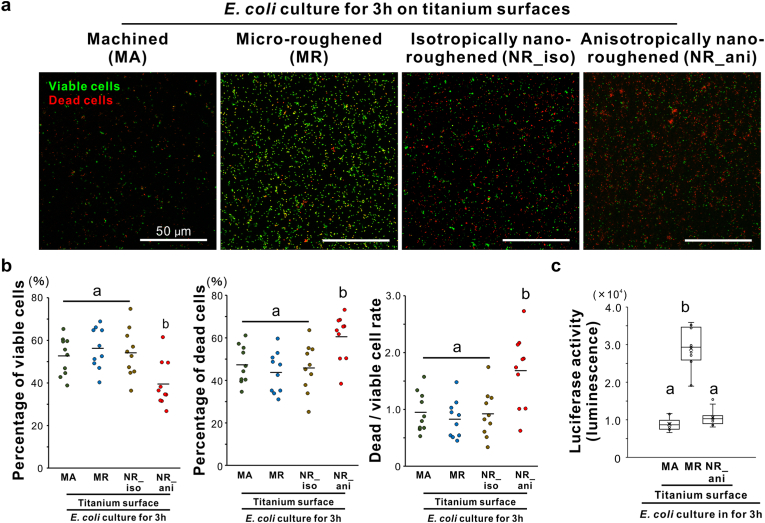
Effects of titanium nanosurface on viability of *Escherichia coli,* Confocal laser microscopy images (a) of *Escherichia coli* Top10 strain (*E. coli*) cultured in brain heart infusion broth for 3 h after live (green) and dead (red) fluorescent staining on machined (MA), micro-roughened (MR), nano-roughened 1 (NR_iso), and nano-roughened 2 (NR_ani) titanium surfaces. Percentages of viable and dead cells and dead/viable ratio measured from the confocal laser microscope images (b), and luciferase activity measuring the amount of bacterial adenosine triphosphate in the culture (c) on each titanium surface. Data are presented as dot plots in (b) (*N* = 10), and box-and-whisker plots with dots and means ( × ) in (c) (*N* = 9). Different letters indicate statistically significant differences (*P* < 0.05; Tukey's honestly significant differences test).

For 3-h *E*. *coli* culture, the broadest fluorescent signals for viable cells were detected on MR surfaces (Fig. 6a). The nanoroughened titanium surfaces exhibited more extensive fluorescent signals than the MA surfaces. However, most of the fluorescent signals on the NR_ani surface indicated dead cells, whereas the viable and dead signals were approximately equal on the other surfaces. The percentages of viable and dead cells on the NR_ani surfaces was approximately 40 % and 60 %, respectively (Fig. 6b). The dead/viable cell ratio was over 1.5 on the NR_ani surfaces in contrast with 1.0 or less on the other surfaces (Fig. 6b). Luciferase activity in the 3-h *E coli* culture was three times higher on the MR surfaces than on the NR_ani surfaces, which was comparable to the value with the MA surfaces (Fig. 6c).

## 4 Discussion

Both types of titanium nanosurfaces consist of densely packed nanospikes (Fig. 1f) with pits between the nanospikes that were smaller than the typical diameter of cocci (Fig. 1h), which is approximately 1.0 μm [39]. Similar to previous studies [15,16,[19], [20], [21], [22],^25^,^26^], this feature might allow physical contact of nanospikes with bacteria without entrapping their cell bodies. In addition, the crystallographic features of the superficial layer (Fig. 2a and b, and S2), atomic composition (Fig. 1e), superhydrophilicity (Fig. 2c), and presence of hydroxyl groups (Fig. 2d) were similar for both types of titanium nanosurfaces. These observations are consistent with our previous reports, which showed that both types of titanium nanosurfaces consist of a hybrid of sodium titanate and brookite-type titanium oxide [36,37,40,41]. These are typical physicochemical surface properties imparted by titanium nanosurface modification via sodium hydroxide–based alkaline etching [41,42]. Moreover, the hydroxyl group is involved not only in hydrophilicity but also in the negative surface potential of titanium oxide [13,43,44]. The alkaline etching–treated titanium surface has apparently negative surface potential [45] in contrast to machined or micro-roughened titanium surfaces, which have a zeta potential of nearly ±0 mV (Fig. 2g). Both the NR_iso and NR_ani particles obtained from the superficial layer had similar isoelectric points and titration plot profiles at subsequent pH values (Fig. 2e). However, the negative zeta potential of the solid surface was more than three times greater for the NR_ani surface than for the NR_iso surface (Fig. 2g). In addition, analysis of the nano-level localization and degree of contact potential difference, which indicates the ease of electron emission, revealed that the value was elevated at the vertices of the nanospikes on both types of titanium nanosurfaces (Fig. 2h). This indicates that electrons are easily released from the vertices of the nanospikes and supports the previously reported speculation that negative charges on the titanium surface gather at the vertices of sharp ridges or protrusions [29]. More importantly, the average value of the NR_ani titanium nanosurfaces was lower; however, the unbiased variance and maximum value were several times higher than that of the NR_iso surfaces (Fig. 2i). These observations indicate that the positional information of the nanospikes on the surface determined the electrical reactivity of the titanium surface.

The differences in properties between the NR_iso and NR_ani surfaces were only nanotopography features, such as vertical surface roughness (thickness of the nano-superficial layer) (Fig. 1, Fig. 2b), vertex density, and anisotropy (Fig. 1f–g). Anisotropy in the two-dimensional distribution pattern of the nanospike vertices was two times higher on the NR_ani than on the NR_iso surfaces (Fig. 1g). The anisotropy of the nanospike distribution on the NR_ani surface was much higher than testimated by the algorithmically derived random pattern model [36]; however, the isotropically patterned nanospikes on the NR_iso surface were lower than that estimated by the model. The vertical roughness of the NR_ani surface was also 1.5–2 times higher than that of the NR_iso surface (Fig. 1i). However, in the particle state, the difference in particle size did not influence the zeta potential between the titanium nanosurfaces (Fig. 2e and f), suggesting that the longitudinal structural differences between the two types of titanium nanosurfaces did not clearly correlate with the changes in electrical reactivity. In contrast, the product of the distribution anisotropy and density of the surface vertices was positively correlated with the maximum contact potential difference and negative zeta potential values (Fig. 2j). Taken together, the dense and anisotropic distribution of the vertices enhances the electrical reactivity of titanium surfaces beyond their inherent properties.

*S. aureus* requires proteins for attachment to biomaterials [46], particularly to flat surfaces with poor properties, such as MA titanium surfaces (Fig. 4a–c). In contrast, on roughened titanium surfaces, the properties influence bacterial attachment beyond that of adsorbed proteins (Fig. 4a–c). The MR titanium surfaces apparently entrapped the cell body in micron pits (Fig. 3b) and promoted the attachment and colonization of gram-positive cocci such as *S. aureus* or *S. sanguinis* (Fig. 3, Fig. 4a,b and g). The NR_ani surface showed vertical roughness values similar to those of the MR surface (Fig. 1i), which is a generally unfavorable surface topography for preventing bacterial attachment [47]. However, the NR_ani surfaces inhibited both the attachment and colonization of *S. aureus* and *S. sanguinis* to levels equivalent to those on the MA surfaces (Fig. 3, Fig. 4). The NR_iso surface did not show any clear inhibitory effect on the attachment and colonization of *S. aureus*, but rather showed a level of bacterial attachment close to that of the MR surface (Fig. 3e and g), despite being relatively flat (Fig. 1i) and without bacteria-entrapping pits (Fig. 1h).

The water contact angle was positively correlated with *S. aureus* attachment to the titanium surfaces (Fig. 4e). Bacteria approach the material surface not only because of the autonomous movement, but also because of external forces from the environment, such as Brownian motion, sedimentation, aggregation, and liquid flow generated by the material's surface free energies [48]. The initial attachment of bacteria is influenced by attractive or repulsive interactions with material surfaces at distances of 50 nm or less, owing to physical forces such as surface free energies [49,50].

It is difficult for hydrophobic bacteria, such as *S. aureus*, to adhere to hydrophilic surfaces [1,6]. *S. aureus* adheres to materials by nonspecific binding of cell wall macromolecules to the material surface, and this process is influenced by the wettability of the surface [51]. Additionally, the electrical properties of material surfaces play an important role in bacterial attachment. The cell walls of gram-positive bacteria are mainly composed of a thick peptidoglycan layer anchored by lipoteichoic and teichoic acids [52]. These components provide the cell wall with a net negative charge [53]. Hence, negatively charged material surfaces can electrostatically repel bacteria [6]. In particular, *S. aureus* colonization of biomaterials is markedly reduced by repulsive electrostatic forces [53]. In this study, the negative zeta potential showed a positive correlation with *S. aureus* attachment to titanium surfaces (Fig. 4f). Lipoteichoic acids are major hydrophobins that bind to hydrocarbons on hydrophobic surfaces [54]. The glycolipid content that determines the hydrophobicity of lipoteichoic acids varies in a genus-specific manner [55]. Therefore, the NR_ani surfaces reduce the attachment and colonization of gram-positive cocci universally through their superhydrophilicity and net negative charge, whereas the anti-biofouling effects of the NR_iso surfaces on cocci adhesion are limited owing to less electric reactivity.

Interestingly, representative gram-negative bacteria, *E. coli* were elongated only on NR_ani titanium surfaces (Fig. 5a); they were two to seven times longer than the general *E coli* size (1 μm) [38]. Cell body elongation of *E. coli* is observed during cell lysis by beta-lactam antibiotics such as cephalexin [56]. This phenomenon is the preliminary stage of cell bursting via bulge formation [57]. The Feret diameter of *E. coli* on the titanium surfaces positively correlated with the electrical properties of the titanium surfaces (Fig. 5c and d). The surface potential of bacteria is closely related to their viability or proliferative activity, and beta-lactam antibiotics exert their antimicrobial effects by disturbing bacterial surface potentials [58]. It was noted that the elongated bacteria on the NR_ani surface died (Fig. 6). Changes in bacterial surface potential are linked to bacterial membrane permeability and can lead to cell death beyond a critical point [59]. The outer membrane layer containing lipopolysaccharides encapsulates gram-negative bacteria, and its integrity is compromised by electrical stimulating agents [60]. Again, the negative electric potential and reactivity of the NR_ani surfaces were much higher than those of the NR_iso surfaces, in association with an increase in the density and anisotropy of the vertex distribution of the titanium oxide nanospikes (Fig. 2, Fig. 5, Fig. 6). These observations suggest that NR_ani surfaces kill *E. coli* by disturbing the bacterial surface potential with electrons released from the vertices of the titanium oxide nanospikes. In contrast, the NR_iso surfaces did not have sufficient electrical reactivity to exert these biological effects.

**Fig. 7:**
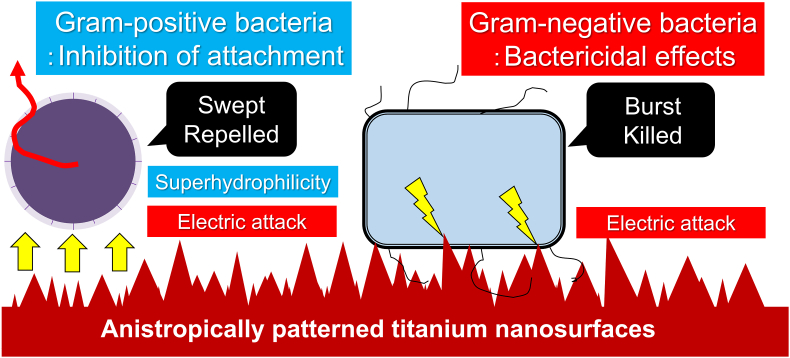
Possible mechanism underlying mechano-bactericidal effects of anisotropically patterned titanium nanosurface, Schematic showing the possible mechanism underlying the mechano-bactericidal effects of anisotropically patterned titanium nanosurfaces. Titanium nanosurfaces with anisotropically patterned dense nanospikes increase the intrinsic electric field strength of the superficial titanium oxide layer owing to the synergistic effect of the anisotropic pattern and density of the titanium nanospikes, thereby enhancing the electrochemical reactivity. The enhanced electrochemical reactivity of the anisotropically patterned titanium nanosurface bounces stiffer gram-negative bacteria with duller reactivity to electrical changes on the cell surface, together with superhydrophilicity. Moreover, this enhanced electrical reactivity bursts and kills softer gram-negative bacteria with high reactivity to electrical stimulation by inhibiting bacterial growth.

The structural rigidity of the bacterial cell wall may be associated with bacterial reactivity on nano-roughened titanium surfaces. Gram-negative bacteria tended to be more negatively charged in surface electric potential (from −20 to −40 mV) than that in gram-positive bacteria (from −15 to −30 mV) [61,62]. This electrical property of gram-negative bacteria is linked to the higher susceptibility of electron acceptors or donors than that of gram-positive bacteria [59,61,63,64]. The peptidoglycan layer of gram-negative bacteria is only a few nanometers thick and is markedly thinner than the 30–100-nm thick peptidoglycan layer of gram-positive bacteria [65]. Peptidoglycan is a large polymer that determines the stress resistance and shape-determining properties of bacterial cell walls [66]. Bacterial stiffness varies depending on cell wall structure, as shown in the previous report that Young's modulus of gram-positive bacteria is over 0.2 N/m and stiffer than under 0.2 N/m stiffness of gram-negative bacteria [67]. In addition, gram-negative bacteria have markedly lower turgor pressure than gram-positive bacteria [[68], [69], [70], [71]], the degree of which is linearly correlated with the difficulty in deformation of the cell body as well as bacterial division [72,73]. Indeed, it has been ultrastructurally demonstrated that titanium oxide nanopillars that physically induce bacterial intracellular reactive oxygen species are more likely to deform the cell bodies of gram-negative bacteria than those of gram-positive bacteria [26]. Taken together, the NR_ani titanium surfaces exerted anti-biofouling or mechano-bactericidal effects against gram-positive or negative bacteria by bouncing or electrically killing cell bodies, respectively (Fig. 7). Moreover, the coexistence of bactericidal properties and superhydrophilicity is favorable for the surface of antibacterial biomaterials because it allows the washing away of dead bacteria, which serves as the starting point for new bacterial growth.

More importantly, materially identical titanium oxide nanospikes enhance their overall electrical properties and alter their biological effects by simply increasing the density and anisotropy of their distribution. This principle of topographic adjustment may maximally enhance the inherent biological properties of various types of conductive or semi-conductive biomaterials. In addition, NR_ani titanium surfaces regulate the function of various types of mammalian cells, such as periodontal ligament cells, gingival fibroblasts, osteocytes, and macrophages, to induce tissue regeneration or anti-inflammation [[34], [35], [36],^40^,^41^] by physically stimulating the cell bodies [36]. None of these biological effects were induced by the NR_iso titanium surfaces. Therefore, this simple titanium nanosurface modification technology and its principles may pave the way for the development of novel implantable biomaterials to simultaneously tune the host/microorganism ecosystem to maintain tissue homeostasis.

## 5 Conclusion

Titanium nanosurfaces with dense and anisotropically patterned titanium oxide nanospikes exert antimicrobial activity against gram-positive cocci and gram-negative bacilli by repelling and killing them, respectively. Other types of titanium surfaces with smooth machined grooves, micro-roughened irregularities, or isotropically patterned nanospikes do not exhibit mechano-bactericidal effects. These antimicrobial capabilities are attributed to superhydrophilicity and electrochemical reactivity in addition to nanosized irregularities finer than those of the bacterial cell body. The dense and anisotropic distribution of nanospikes amplifies the inherent electrochemical reactivity and disturbs the bacterial cell surface.

## Funding sources

This work was supported by a Grant-in-Aid for Scientific Research (B: 24K02625, M.Y. and H.E.) and a Grant-in-Aid for Challenging Research (Exploratory) (23K18585, M.Y. and H.E.) from the Japan Society for the Promotion of Science and Tokyo Dental College Branding Project for Multidisciplinary Research Center for Jaw Disease from the Ministry of Education, Culture, Sports, Science and Technology (MEXT).

## CRediT authorship contribution statement

**Eiji Kato:** Writing – original draft, Visualization, Methodology, Investigation, Formal analysis. **Masahiro Yamada:** Writing – review & editing, Methodology, Investigation, Funding acquisition, Formal analysis, Conceptualization. **Eitoyo Kokubu:** Writing – review & editing, Methodology. **Hiroshi Egusa:** Writing – review & editing, Funding acquisition. **Kazuyuki Ishihara:** Writing – review & editing, Supervision, Funding acquisition, Formal analysis.

## Declaration of competing interest

The authors declare that they have no known competing financial interests or personal relationships that could have appeared to influence the work reported in this paper.

## Data Availability

Data will be made available on request.
