# Supplementary material for: Anisotropic patterns of nanospikes induces anti-biofouling and mechano-bactericidal effects of titanium nanosurfaces with electrical cue
Source: Mater Today Bio. 2024 Nov 22;29:101352. doi: 10.1016/j.mtbio.2024.101352 (PMC11636339; doi:10.1016/j.mtbio.2024.101352)
Supplement: Multimedia component 1 [file mmc1.docx]

**Anisotropic patterns of nanospikes induces anti-biofouling and mechano-bactericidal effects of titanium nanosurfaces with electrical cue**

*Eiji Kato^a,b^, Masahiro Yamada^c^*, Eitoyo Kokubu^1^, Hiroshi Egusa^c, d^ and Kazuyuki Ishihara^a^.*

^a^ Department of Microbiology, Tokyo Dental College, Tokyo, 101-0061, Japan

^b^ Implant & Tissue Engineering Dental Network-Tokyo, 153-0051, Tokyo, Japan

^c^ Division of Molecular and Regenerative Prosthodontics, Tohoku University Graduate School of Dentistry, Sendai, Miyagi, 980-8575, Japan.

^d^ Center for Advanced Stem Cell and Regenerative Research, Tohoku University Graduate School of Dentistry, Sendai, Miyagi, 980-8575, Japan.

**Supplementary Material and methods**

*1. Preparation of nano-roughened titanium samples*

The cleaned machined (MA) titanium samples were boiled in a 5 or 10 M sodium hydrate solution for 24 h at 60 or 90 °C, respectively. After boiling, the titanium samples were washed with DW and dried overnight in the dark under ambient conditions. The titanium samples were then sintered in a furnace with a linearly increasing temperature of 5 °C/min up to 600 °C, with the final temperature maintained for 1 h, followed by natural cooling after sintering. Boiling with a 5 or 10 M sodium hydrate solution provided different nano-roughened (NR) titanium surfaces, namely, NR_iso and NR_ani, respectively.

*2. Zeta potential and particle size measurements*

To measure the isoelectric point, a nano-particle suspension of material comprising a superficial layer of NR_iso or NR_ani titanium sheets was prepared by scraping the sheet surface in distilled water with a glass scraper. After allowing the large particles and glass shards to settle naturally for 24 h, the collected supernatant was used as the nanoparticle suspension of the titanium nanosurface. The zeta potential of the nanoparticles was determined from the electrophoretic mobility based on the Smolkowski equation. The pH titration plot of the nanoparticle suspension was created by adjusting the pH within the range of pH 2.5 to 7.0 using 0.1 mol/L sodium hydroxide and hydrochloric acid and measuring the zeta potential at each pH point. In the pH titration plot, the pH at which the zeta potential reached zero was determined to be the isoelectric point of each titanium nanosurface. In addition, the particle size distribution in the suspension was determined using dynamic light scattering.

To measure zeta potential of the titanium surfaces, a colloidal solution of hydroxypropyl cellulose–coated polystyrene latex with a particle size of approximately 500 nm and almost zero surface potential, adjusted to pH 6.0, was placed inside a chamber with a titanium sheet at the bottom. After applying an electric field of -1.53 V/cm inside the chamber, the apparent electrophoretic mobility of the particles was measured, and the electroosmotic flow was analyzed using the Mori–Okamoto equation to calculate the zeta potential on the surface of the titanium sheet.


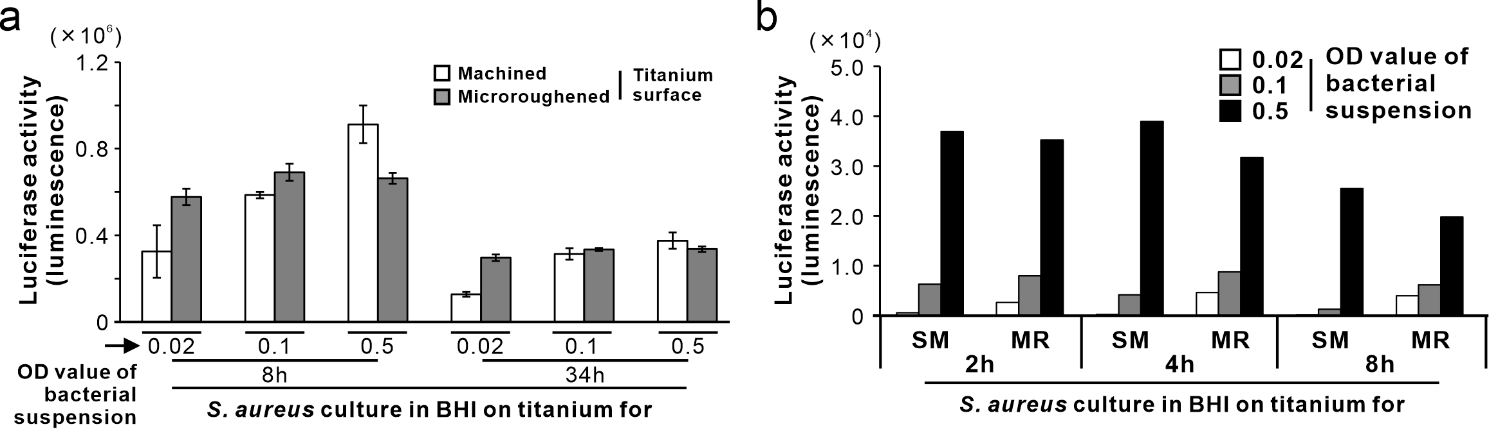


**Figure S1. Determination of *Staphylococcus aureus* 209P strain culture condition on titanium surfaces**

(a) Luciferase activity measuring the amount of bacterial adenosine triphosphate in the *Staphylococcus aureus* 209P strain cultured in brain heart infusion (BHI) broth bacterial suspension at an optical density (OD) of 0.02, 0.1, or 0.5 for 8 and 34 h on machined (MA) and micro-roughened (MR) titanium surfaces. (b) Luciferase activity in *S. aureus* culture in BHI broth bacterial suspension at an OD of 0.02, 0.1, or 0.5 for 2, 4, and 8 h on MA and MR titanium surfaces. Data are presented as means ± standard deviation (SD) in (a) (*N* = 4) and histograms without SD in (b) (*N* = 1).


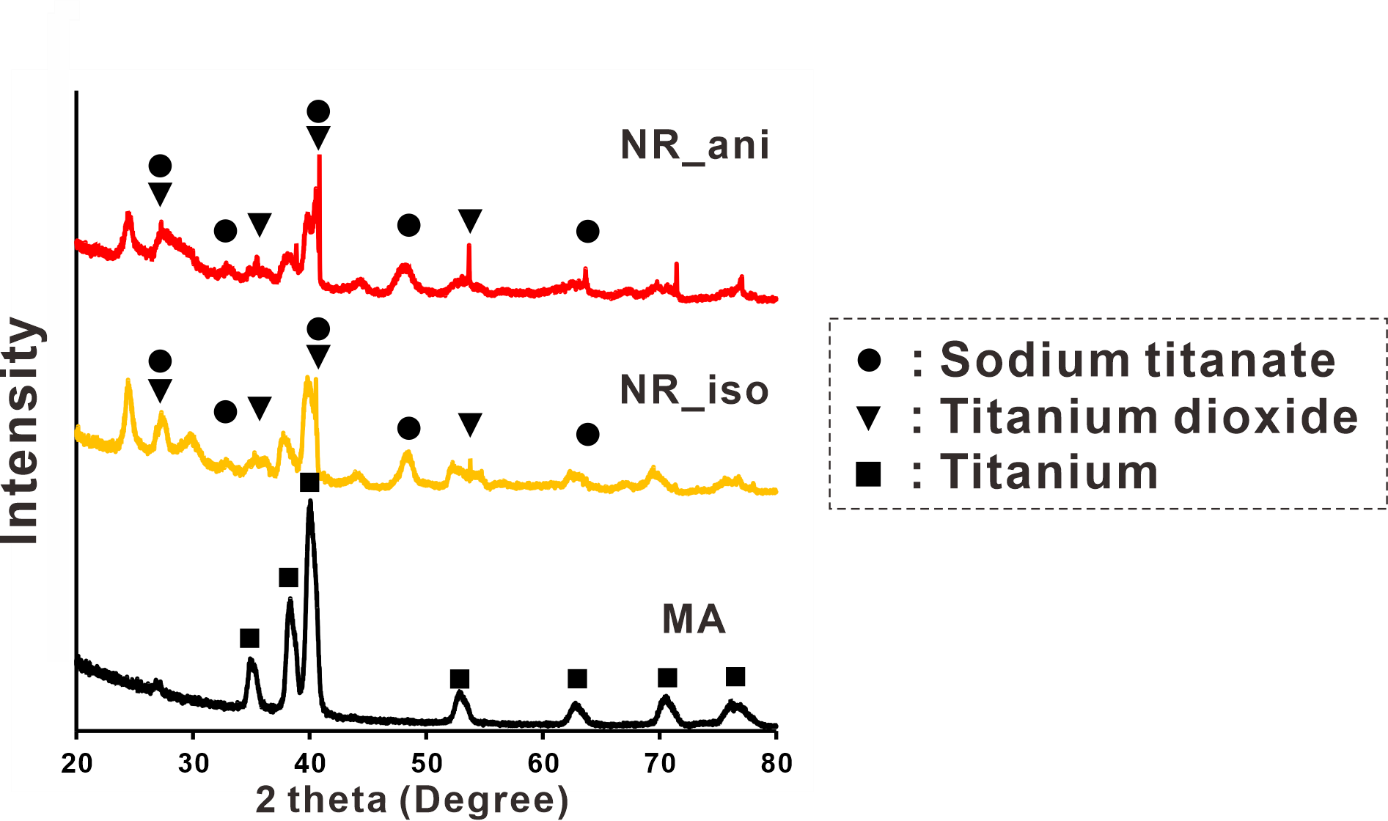


**Figure S2. Crystalline phase of titanium nanosurfaces**

X-ray diffraction (XRD) spectra of titanium discs with machined, and isotropically nanoroughened (NR_iso) and anisotropically nanoroughened (NR_ani) surfaces. The circles, triangles, and squares in the XRD patterns indicate peaks corresponding to sodium titanate, titanium dioxide, and titanium, respectively.
